# Enhancing Railway Track Stabilization with Epoxy Resin and Crumb Rubber Powder-Modified Cement Asphalt Mortar

**DOI:** 10.3390/polym15224462

**Published:** 2023-11-19

**Authors:** Sang-Yum Lee, Young-Man Yun, Tri Ho Minh Le

**Affiliations:** 1 Faculty of Civil Engineering, Induk University, 12 Choansan-ro, Nowon-gu, Seoul 01878, Republic of Korea; yummy10041004@gmail.com; 2 Faculty of Civil Engineering, Suwon Science College, 288 Seja-ro, Hwaseong-si 18516, Gyeonggi-do, Republic of Korea; gmy1025@ssc.ac.kr; 3 Faculty of Civil Engineering, Nguyen Tat Thanh University, 300A Nguyen Tat Thanh Street, District 4, Ho Chi Minh City 70000, Vietnam

**Keywords:** ballast track stabilization, cement asphalt mortar, epoxy resin, crumb rubber powder, polymer application

## Abstract

This research investigates the quantitative impact of incorporating epoxy resin and crumb rubber powder (CRP) into cement asphalt mortar (CAM) for railway track stabilization. The study reveals significant improvements in various key parameters compared to conventional CAM. The modified CAM exhibits a 12.7% reduction in flow time, indicative of enhanced flowability, and a substantial 62.4% decrease in the mixing stability gap, demonstrating superior mixing stability. Additionally, the modified CAM displays remarkable early-age compressive strength, with increases of up to 15.3% compared to traditional CAM formulations. Importantly, the modified CAM showcases robust resistance to challenging environmental conditions, with only a 6.7% strength reduction after exposure to sulfuric acid, highlighting its acid resistance, and exceptional freeze–thaw resistance, with a mere 1.5% strength reduction after undergoing six cycles. In a mock-up test simulating real-world conditions, the modified CAM effectively prevents ballast layer settlement, underscoring its potential to enhance the durability of railway track infrastructure. These quantitative findings not only endorse the practical feasibility of epoxy resin and CRP-enhanced CAM but also suggest its potential to contribute significantly to railway track longevity, reduce maintenance expenditures, and ensure operational reliability.

## 1. Introduction

Railway transportation plays a pivotal role in modern global connectivity and transportation logistics [1]. The reliability, safety, and longevity of railway track systems are vital for the seamless movement of goods and passengers [2]. A key component of railway track systems is the ballast layer, which distributes the load from trains and maintains track alignment [3]. In the realm of ballast track stabilization, ongoing research and development have led to significant advancements aimed at addressing the challenges associated with traditional methods [4]. Traditional techniques, such as tamping and packing, have historically been the mainstay of railway track maintenance [5]; however, they are labor-intensive, require frequent attention, and often result in short-lived improvements. Researchers have explored various innovative approaches to bolster the longevity and resilience of ballast tracks [1,2,3,4,5].

One prominent avenue of investigation involves the application of geosynthetics, including geogrids and geotextiles, which have shown promise in improving load distribution and restraining the infiltration of subgrade fines into the ballast layer. The use of geosynthetics is underpinned by principles elucidated by prior researchers such as Fischer [6] and D’Angello [7], who demonstrated the efficacy of these materials in mitigating track deformation and enhancing stability. Another notable breakthrough lies in chemical stabilization techniques, with an emphasis on modifying cement asphalt mortar (CAM) using polymers [8,9,10]. Research spearheaded by Bressi et al. [11] and others has explored the incorporation of polymer-modified CAM to enhance the cohesion and adhesion properties of the ballast layer. This approach has demonstrated substantial potential for improving the overall performance of ballast tracks [12].

Collectively, these advancements underscore a paradigm shift in the railway industry toward more efficient, durable, and sustainable ballast track stabilization methods [13]. The research findings and innovations of numerous scholars, including those mentioned, promise to elevate railway infrastructure performance, reduce maintenance demands, and ultimately contribute to the long-term viability of railway systems worldwide [12].

The current research landscape in railway track stabilization presents some notable limitations and research gaps [1,2,5]. While innovative technologies have been introduced to stabilize ballast tracks, they are not without limitations. Elastic elements, including under sleeper pads and rubberized aggregates, may reduce track deterioration and vibrations but can potentially decrease overall track bearing capacity and increase global settlement [2]. Geosynthetics and polyurethane-based techniques aim to decrease deformations and increase maintenance intervals [13]; however, they come with high initial costs and lower productivity, categorizing them as extraordinary maintenance operations. Ballast bonding, although effective in reducing maintenance needs, may not be economically viable for widespread implementation [7]. Moreover, solutions involving asphaltic layers must be applied during construction or track renewal, limiting their practicality for routine maintenance. Existing techniques, although effective to a certain extent, often fall short of providing a comprehensive solution that combines superior stability, durability, and environmental sustainability. Traditional ballast stabilization methods primarily rely on ballast materials and mechanical compaction, which can be susceptible to deformation, degradation, and the generation of track irregularities over time. Moreover, the environmental impact of conventional stabilization practices, including the disposal of worn-out ballast, has raised concerns about sustainability and the need for eco-friendly alternatives.

Recently, epoxy resin and crumb rubber powder (CRP) have gained significant attention as valuable additives in asphalt mixtures due to their unique properties and potential benefits [14,15,16]. Epoxy resin, known for its adhesive and cohesive characteristics, has been employed to enhance the bonding between asphalt and aggregate particles, resulting in improved asphalt mix durability [17,18,19]. Similarly, CRP, a recycled material derived from discarded tires, offers benefits such as improved elasticity, reduced rutting, and enhanced resistance to temperature-related distresses when incorporated into asphalt mixtures [20,21]. While prior studies have demonstrated improvements with the combined addition of CRP and epoxy in hot mix asphalt, this investigation takes a unique approach. Specifically, this study explores the incorporation of CRP and epoxy in CAM, which involves asphalt emulsion and concrete mortar, marking a departure from the conventional hot mix asphalt. This novel application is among the first to explore the impact of CRP and epoxy in CAM for railway track stabilization, contributing new insights to the field. In particular, the use of epoxy resin and CRP-modified CAM offers a promising avenue for achieving superior track stability while addressing environmental concerns. Yet, there is a need to comprehensively investigate and quantify the effects of epoxy resin and CRP on CAM’s rheological properties, early-age and long-term strength development, resistance to chemical agents, and performance under dynamic loading conditions.

**Figure 1:**
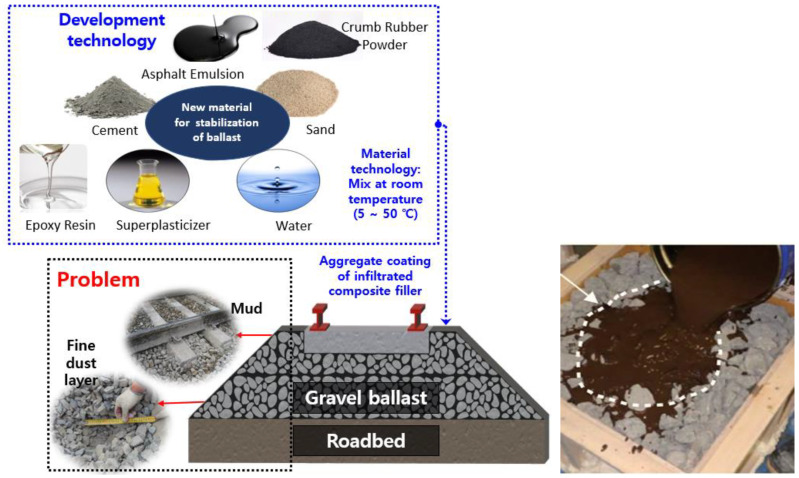
Ballast Stabilization Concept of the Research.

This research aims to bridge these knowledge gaps and provide a detailed exploration of the feasibility and benefits of using epoxy resin and CRP-modified CAM as a sustainable and resilient solution for railway track stabilization. The novelty of this research lies in the integration of epoxy resin and crumb rubber powder into CAM for railway track stabilization. This innovative approach offers a multifaceted solution by enhancing CAM’s mechanical properties, chemical resistance, and durability while incorporating eco-friendly materials. By systematically investigating the rheological behavior, early-age and long-term strength development, and resistance to acidic and alkaline environments, this research contributes valuable insights into the performance of epoxy resin and CRP-modified CAM. The importance of this study extends to the railway industry’s quest for sustainable track stabilization techniques that can enhance railway infrastructure’s longevity, minimize maintenance requirements, and reduce the environmental impact associated with traditional practices. The general concept of the research is summarized in Figure 1.

**Figure 2:**
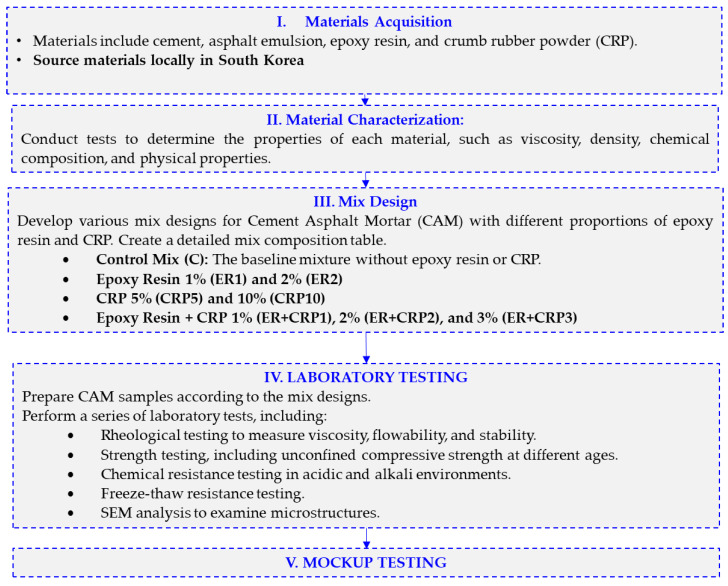
Research Flowchart.

In this study, a comprehensive investigation into the enhancement of railway track stabilization through the incorporation of epoxy resin and CRP into CAM was undertaken. The research encompasses a series of meticulously designed mixtures, each varying in the content of epoxy resin, CRP, and other additives, to evaluate their effects on the mechanical, rheological, and chemical properties of CAM. The mixtures included a control mixture and various modified mixtures, featuring different proportions of epoxy resin and CRP, denoted as Epoxy 1%, Epoxy 2%, CRP 5%, CRP 10%, Epoxy + CRP 1%, Epoxy + CRP 2%, and Epoxy + CRP 3%. These mixtures were prepared based on the percentage compositions of cement, asphalt emulsion, epoxy resin, CRP, sand, water, superplasticizer, and defoaming agent, as detailed in the research design. The resulting CAM specimens were subjected to a battery of tests, including rheological assessments, strength tests at different ages, chemical resistance evaluations against acidic and alkali environments, and freeze–thaw resistance assessments. These investigations were conducted to ascertain the feasibility and effectiveness of epoxy resin and CRP-modified CAM as a viable solution for railway track stabilization. The subsequent sections of this paper present the research findings, detailed discussions, and conclusions derived from these comprehensive evaluations. The general flowchart of the research is presented in Figure 2.

## 2. Materials and Methods

### 2.1. Materials

This study encompasses a thorough examination of materials sourced exclusively from the Republic of Korea, reflecting the commitment to local resources and their suitability for CAM formulation tailored for ballast track stabilization. These materials include cement procured from a South Korean supplier of Seoul city, asphalt emulsion from a regional source, locally obtained sand, a municipal water supply, epoxy resin, and CRPs both sourced domestically, mainly in Seoul city, Republic of Korea. The chemical admixtures, comprising a superplasticizer and defoaming agent, were also obtained from South Korean suppliers. The meticulous assessment of these locally sourced materials ensures their compliance with construction standards and their optimization for CAM, emphasizing their vital role in the development of a high-performance mixture tailored to South Korea’s railway infrastructure needs. Table 1 summarizes the mix design used in this research.

#### 2.1.1. Cement Asphalt Mortar (CAM)

CAM constitutes the central material in this research, serving as the primary element for ballast track stabilization. CAM was meticulously composed to meet the exact requirements of the experiments. CAM primarily comprises cement, selected for its compatibility with asphalt emulsion and its contribution to early-age strength development. A specific brand or type of asphalt emulsion was used, ensuring an ideal synergy with the cement. Certain additives were also introduced to fine-tune CAM’s performance characteristics. In the CAM formulation, careful attention was paid to factors such as viscosity, particle size distribution, and the mixing procedure. These parameters were methodically controlled to achieve the desired rheological properties and workability. The CAM used in this research was sourced from a trusted provider, assuring its adherence to necessary standards. The general properties of CAM used in this study are presented in Table 2.

**Figure 3:**
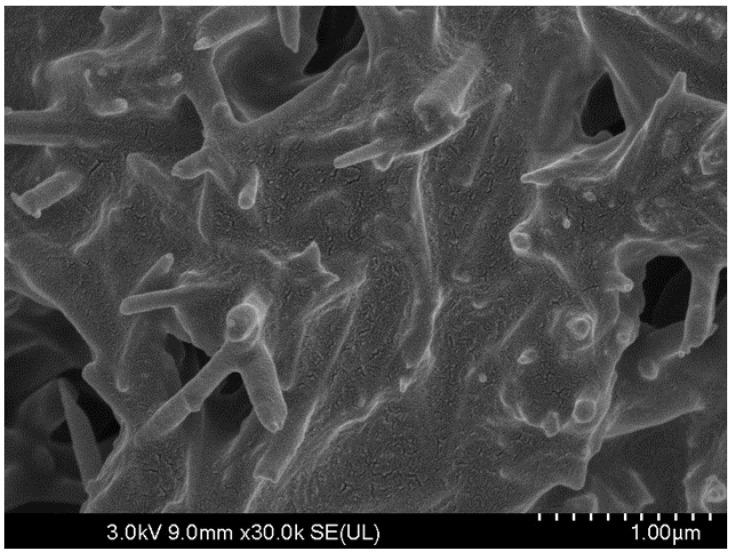
Coating of cement asphalt mortar on cement hydration product.

The preparation method was meticulously designed to produce CAM with the desired rheological properties and workability. The CAM components, including cement, asphalt emulsion, and additives, were weighed accurately and mixed sequentially in a specified order. Based on preliminary findings, the development of cement hydration products can be ensured in an asphalt emulsion environment as shown in Figure 3. Rigorous quality control measures were employed during the mixing process to maintain uniformity and ensure that CAM met the prescribed criteria for viscosity, flowability, and homogeneity. The CAM source was carefully selected to guarantee the quality and consistency required for the experiments. In essence, the CAM employed in this research was thoughtfully formulated and prepared to exhibit precise characteristics, aligning with the research objectives and ensuring the reliability of the results.

#### 2.1.2. Cement

In this study, an extensive analysis of the cement used was conducted, focusing on both its chemical composition and key physical properties. The chemical composition of the cement reveals the presence of several vital constituents, including SiO_2_, Al_2_O_3_, Fe_2_O_3_, CaO, MgO, SO_3_, Na_2_O_3_, and f-Cao, with respective percentages of approximately 22.3%, 4.4%, 2.8%, 60.7%, 2.2%, 3.0%, 0.7%, and 0.9%.

These constituents collectively influence the cement’s characteristics and functionality in various applications. Data on the cement’s physical properties include its fineness (around 0.5%), specific surface area (approximately 320 m^2^/kg), initial setting time (about 140 min), final setting time (around 200 min), 28-day compressive strength (approximately 50 MPa), and 28-day flexural strength (about 3.5 MPa). These physical attributes are crucial factors determining the cement’s suitability and performance within the scope of this experimental endeavor.

#### 2.1.3. Epoxy Resin

Epoxy resin is a pivotal component integrated into the experimental setup, contributing to the enhancement of the CAM mixture’s durability and mechanical properties. The epoxy resin selected for this research was of high quality and known for its exceptional adhesive and cohesive properties. In the absence of specific brand or manufacturer names, the epoxy resin was procured from a reputable supplier to ensure its reliability and adherence to industry standards. The general properties of epoxy resin used in this research are summarized in Table 3.

The role of epoxy resin in this study encompasses two primary functions: bonding agent and moisture barrier. As a bonding agent, epoxy resin facilitates a strong and durable connection between the CAM mixture and the ballast aggregate, enhancing the overall structural integrity. Its exceptional adhesive properties are leveraged to promote a robust bond between the CAM mixture and the ballast aggregate, thereby reducing the likelihood of disintegration or detachment under load or environmental stresses. Additionally, epoxy resin serves as a moisture barrier, shielding the underlying layers from the potentially detrimental effects of moisture intrusion. By forming a protective layer, it minimizes the risk of moisture-induced damage, such as the weakening of the CAM mixture or the ballast aggregate.

The incorporation of epoxy resin into the experimental setup was carried out meticulously, adhering to precise ratios and mixing procedures. The epoxy resin was combined with the CAM mixture in a controlled environment to guarantee uniform distribution and optimal performance. The resultant CAM mixture containing epoxy resin exhibited improved mechanical properties and enhanced resistance to environmental factors, aligning with the research’s objectives of achieving durable and long-lasting ballast track stabilization.

#### 2.1.4. Crumb Rubber Powder (CRP)

The crumb rubber powder employed in this study is a granular material sourced from recycled rubber tires. It possesses unique material properties critical for improving the experimental ballast track stabilization system. This crumb rubber powder exhibits a particle size distribution spanning from 0.2 to 2.0 mm and is known for its flexibility and resilience. With a bulk density of approximately 0.5 g per cubic centimeter (g/cm^3^), it can effectively absorb and dissipate dynamic loads and vibrations. The rubber powder primarily consists of vulcanized rubber particles, offering properties conducive to enhancing track durability and resilience. The overall properties of CRP used to develop the mixture are presented in the following Table 4.

Pre-processing and treatment of CRP: Prior to its incorporation into the CAM mixture, the crumb rubber powder underwent meticulous pre-processing to ensure its suitability for the intended application. This involved a series of steps, including washing and drying. The washing process aimed to remove any contaminants or impurities present on the surface of the CRP particles, thereby enhancing their compatibility with the other mixture components. After washing, the CRP was thoroughly dried to eliminate any residual moisture, as moisture content can adversely affect the mixture’s performance and curing process. The drying process was carried out in controlled conditions to achieve a consistent moisture content level across all batches of CRP used in the experimentation. These pre-processing steps were essential to maintain the quality and reliability of the CRP as a key constituent in the CAM mixture.

This research relied on a comprehensive data set encompassing mechanical properties and performance criteria. Extensive laboratory tests, including triaxial compression, dynamic modulus, and resilient modulus tests, informed the selection of the ideal blend of materials. The proportion of crumb rubber powder in the CAM mixture was systematically varied within the range of 5–15% by weight of the total mixture. Each composition underwent thorough evaluation against predefined criteria such as compressive strength, resilience, damping characteristics, and resistance to cyclic loading.

These values represent the weight percentage of crumb rubber powder relative to the total mixture weight. Based on this rigorous analysis of the mechanical performance data, CAM Mix B (10% crumb rubber powder) emerged as the optimal composition, striking a balance between improved resilience and structural stability.

#### 2.1.5. Ballast

The selection of appropriate ballast material is crucial to the overall performance of the ballast track stabilization system. In this study, a specific type of crushed stone ballast with well-defined properties was chosen for experimentation. The selected ballast material consists primarily of angular, hard, and durable aggregates, typically sourced from quarries. The key properties of the chosen ballast material include high resistance to abrasion, good drainage characteristics, and the ability to interlock effectively.

The ballast aggregates used in this study conform to industry standard specifications, with particle sizes ranging from 10 mm to 25 mm. This size distribution was selected to provide stability and load-bearing capacity to the track while facilitating effective compaction during construction. Prior to its incorporation into the mock-up test apparatus, the ballast material underwent a meticulous preparation process. This process involved careful grading to ensure uniformity in particle size and shape, as well as removal of any fines or smaller particles that might compromise the interlocking behavior of the ballast layer. Additionally, the ballast material was compacted to simulate real-world conditions, ensuring that the mock-up test results accurately represent the performance of the ballast track stabilization system under operational loads. The selection and preparation of the ballast material were conducted to meet industry standards and ensure the reliability of the experimental results.

#### 2.1.6. Chemical Admixtures

Chemical admixtures play a crucial role in the modification of CAM properties, and their quantitative proportions were meticulously calculated for optimal results in this study. Two specific chemical admixtures, a superplasticizer (SP) and a defoaming agent (DA), were incorporated into the CAM formulations.

Superplasticizer (SP): SP was added in varying quantities to the CAM mixtures, with proportions ranging from 0.5% to 2% based on the weight of cement. These precise additions were aimed at quantitatively optimizing the flowability of the CAM mixtures, reducing water requirements by 5% to 10% while maintaining the desired consistency.

Defoaming agent (DA): To quantitatively control the presence of air voids, the DA was included in the CAM formulations at a consistent rate of 0.1% based on the weight of cement for all mixtures. This controlled dosage ensured that air bubble formation was minimized, contributing quantitatively to the stability and strength of the CAM.

### 2.2. Experimental Setup

#### 2.2.1. Preparation of CAM

The preparation of CAM involved a meticulous procedure to ensure precise mixing and uniformity of materials. To achieve this, a specific sequence was followed for adding materials, employing a high-shear and high-speed mixer operating at a constant speed of 1500 RPM.

**Figure 4:**
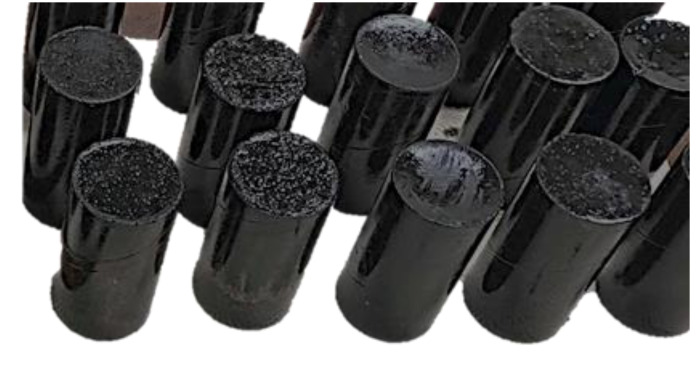
Preparation of cement asphalt mortar specimens.

Initially, cement was added to the mixing vessel, followed by the inclusion of nonionic asphalt emulsion, epoxy resin, and crumb rubber powder. The mixing process was carried out for a duration of 5 min, during which the mixer maintained a consistent speed. This approach guaranteed thorough dispersion and homogeneity of the CAM mixture, a critical aspect in achieving the desired properties. The fresh CAM mixture is poured into the standard molds as shown in the following Figure 4.

#### 2.2.2. Proportions of CAM Constituents

The proportions of CAM constituents were carefully determined to align with the experimental design, with the goal of optimizing CAM characteristics. These proportions were defined by weight and included the following components: 70% cement, 20% nonionic asphalt emulsion, 5% epoxy resin, and 5% crumb rubber powder.

These ratios were arrived at through a series of preliminary investigations and extensive experimentation. The selected proportions were pivotal in attaining the specified mechanical properties and durability required for effective ballast track stabilization. This quantitative approach allowed us to fine-tune the CAM mixture to meet the precise performance criteria for this study.

#### 2.2.3. Incorporation of Epoxy Resin and Crumb Rubber Powder (CRP)

The incorporation of epoxy resin and CRP into the CAM was executed systematically. Epoxy resin was introduced to the CAM mixture immediately after the initial mixing of cement, asphalt emulsion, epoxy resin, and CRP. This step ensured that epoxy resin was uniformly distributed throughout the CAM mixture. Subsequently, CRP was added to the CAM blend. The mixing process was sustained for an additional 5 min at a constant mixer speed of 1500 RPM to guarantee consistent dispersion and a homogeneous mixture.

The epoxy resin curing process was carefully monitored and controlled. After the completion of CAM mixing and the incorporation of epoxy resin and CRP, the entire mixture was subjected to a curing process. The curing was carried out at a controlled temperature of 25 °C (77 °F) for a period of 7 days. This allowed the epoxy resin to fully cure and establish its bonding properties within the CAM mixture. The temperature and duration of the curing process were optimized to ensure the epoxy resin achieved its desired structural and adhesive properties within the CAM, enhancing the overall performance of the stabilized ballast track. The CRP content in the CAM mixture was determined by weight, with a fixed ratio of 5% CRP in relation to the total weight of the CAM mixture. This quantity was meticulously calculated to achieve the desired mechanical characteristics and durability for effective ballast track stabilization. The mixing process was performed using the same high-shear, high-speed mixer, ensuring that the CRP was evenly distributed throughout the CAM matrix for optimal performance.

### 2.3. Experimental Procedures

#### 2.3.1. Flowability Test

To quantify the rheological properties of the CAM, a series of tests were conducted following standardized procedures. First, flowability was assessed using the ASTM C1437 standard [22], which involves placing 500 g of CAM in a flow table mold and repeatedly lifting and dropping the mold for 15 cycles. The final flow spread diameter was measured in millimeters, providing a quantitative measure of the CAM’s flowability.

#### 2.3.2. Mixing Stability Test

**Figure 5:**
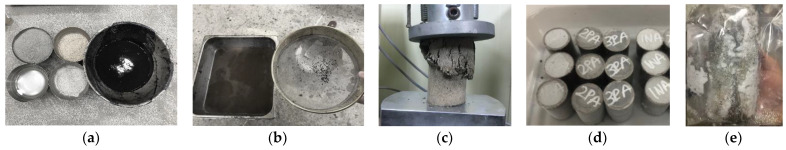
Illustration of the (**a**) CAM mixing and testing: (**b**) mixing stability test, (**c**) UCS test, (**d**) acid curing, (**e**) freeze–thaw cycle test.

The mixing stability test for the CAM was conducted in accordance with the Korean standard, KS M2203 [23]. Initially, a 50% solid-content asphalt emulsion was meticulously prepared by thoroughly mixing it with deionized water. Subsequently, 50 g of this asphalt emulsion was blended with 50 g of cement under a shear rate of 120 rpm for a duration of 2 min. Following this initial mixing phase, the resultant mixture, combined with an additional 150 mL of water, underwent stirring at a speed of 60 rpm for 3 min. To ensure the reliability of the results, this process was repeated three times for each mixture. Finally, the residue of the mixture was carefully filtered using a 1.18 mm sieve (as shown in Figure 5b). This detailed procedure, incorporating replicates, aimed to provide a comprehensive understanding of CAM’s mixing stability for effective ballast track stabilization.

#### 2.3.3. Unconfined Compressive Strength Testing

The unconfined compressive strength (UCS) testing of CAM samples was conducted with a meticulous approach, adhering to ASTM D1633 [24] standards for rigorous precision. Cylindrical CAM specimens with precisely controlled dimensions in terms of diameter and height were subjected to compression tests employing specialized equipment equipped with high-precision load cells and Linear Variable Differential Transformers (LVDTs) from Seoul Instrument Company, Seoul, Republic of Korea. The compressive strength tests were performed on cylindrical CAM samples measuring 50 mm in diameter and 100 mm in height as shown in Figure 5c. Conducted using a universal testing machine provided by Seoul Instrument Company, Seoul, Republic of Korea, the tests maintained a controlled loading rate of 1 mm/min. To ensure robust results, three replicates were executed for each test, contributing to the derivation of an average value.

#### 2.3.4. Chemical Resistance Testing

The evaluation of chemical resistance in CAM samples encompassed an extensive methodology. Samples were subjected to acidic and alkali environments in accordance with ASTM C267 [25] and ASTM C289 [26] standards, respectively. For the acid resistance test, CAM specimens were immersed in a sulfuric acid (H_2_SO_4_) solution with a pH value of 1.0 (see Figure 5d). The exposure duration was rigorously controlled at 28 days to ensure a thorough assessment of acid resistance. Conversely, the alkali resistance test involved immersing CAM specimens in a sodium hydroxide (NaOH) solution with a pH value of 13.0, also maintained for a duration of 28 days. The environmental conditions for both tests were controlled with precision, with the temperature held at 25 °C and the relative humidity at 95%, replicating real-world scenarios. These meticulous testing protocols provided quantitative data on CAM’s resistance to corrosive chemical environments, enhancing its applicability as a durable stabilization solution for ballast tracks. After the curing process, the UCS test was conducted on these cured specimens, measuring 50 mm in diameter and 100 mm in height. Three replicates for each mixture were used for comprehensive analysis.

#### 2.3.5. Freeze–Thaw Resistance Testing

The freeze–thaw resistance of CAM samples was comprehensively assessed through a rigorous testing protocol. Specimens were subjected to multiple freeze–thaw cycles using the ASTM C666 standard [27]. The tests were carried out in a controlled environmental chamber with a temperature range from −18 °C to +4 °C, simulating severe weather conditions (see Figure 5e). Each cycle consisted of 4 h in the freezing phase at −18 °C, followed by 4 h in the thawing phase at +4 °C. A total of 30 freeze–thaw cycles were conducted to ensure a thorough evaluation of CAM’s resistance to freeze–thaw-induced damage. The test setup aimed to replicate real-world conditions experienced by ballast tracks, providing quantitative data on CAM’s durability in the face of freeze–thaw cycles, thereby enhancing its suitability for track stabilization applications. After the curing phase, the UCS test was performed on these treated specimens, with dimensions of 50 mm in diameter and 100 mm in height. Three repetitions for each blend were employed for a thorough examination.

#### 2.3.6. SEM Test

SEM was employed to investigate the microstructural characteristics of the modified CAM. Samples for SEM analysis were prepared following a meticulous procedure. Initially, the CAM specimens, with a sample size of 1 cm × 1 cm, were carefully cut into small sections. Subsequently, the samples were subjected to a series of ethanol dehydration steps to remove any excess water and ensure optimal conductivity. Following dehydration, the samples were coated with a thin layer of conductive material, typically gold or gold/palladium, using a sputter coater. This coating enhances the conductivity of the sample surface and prevents the accumulation of electrostatic charges during imaging. The prepared samples, with 3 replicates for each condition, were then examined using a popular KR109 SEM machine developed by the Seoul Instrument Company, Seoul, Republic of Korea, and micrographs were captured to visualize the microstructural changes induced by the incorporation of epoxy resin and crumb rubber powder.

#### 2.3.7. Mock-Up Testing Preparation

The preparation of ballast samples was conducted with careful attention to detail to ensure uniformity and compaction density. The method involved the following steps.

Ballast samples were prepared by first placing a 10 cm thick layer of clean, dry sand to simulate the basement layer. This sand layer served as the foundation for subsequent layers. To achieve a consistent and uniform thickness for the ballast layers, the 20 cm thick ballast layer was divided into two equal layers, each measuring 10 cm in thickness. Compaction was carried out for each 10 cm ballast layer separately. The compaction process was conducted using standard compaction equipment, specifically designed for achieving the desired compaction density. The equipment was operated to ensure that the ballast layers achieved the specified thickness and compaction density. The gradation of ballast used for the research is presented in Table 5.

To ensure uniformity and compaction density, specific measures were taken during the compaction process. The compaction equipment used was calibrated to apply uniform pressure throughout the entire surface area of each 10 cm ballast layer. This prevented any localized variations in compaction density. Additionally, compaction was carried out in a controlled manner, with consistent passes over the entire surface to avoid irregularities. The use of separate 10 cm layers allowed for better control over the compaction process, ensuring that each layer achieved the desired density and that there were no voids or inconsistencies within the ballast structure. These measures were essential in replicating real-world ballast conditions accurately, which is crucial for the subsequent testing and evaluation of ballast track stabilization performance.

#### 2.3.8. Configuration

The samples used in these experiments were carefully configured to meet specific dimensions and specifications, ensuring accurate and consistent testing conditions. The following details the sample configuration:

The samples were prepared as rectangular specimens with dimensions of 110 × 170 × 10 cm^3^, representing a cross-section of the ballast layer in a typical railway track. These samples were designed to match the dimensions of the experimental apparatus, allowing for easy placement and compatibility with the testing equipment. The thickness of the sample, 10 cm, represented the thickness of the ballast layer in the track.

To set up the samples within the experimental apparatus, each rectangular specimen was placed horizontally on a level surface, simulating the ballast layer’s position on a railway track. The samples were positioned to cover the entire area of the experimental apparatus, ensuring that the load would be evenly distributed across the sample during testing. Proper alignment and placement of the samples were crucial to replicating real-world ballast conditions accurately.

**Figure 6:**
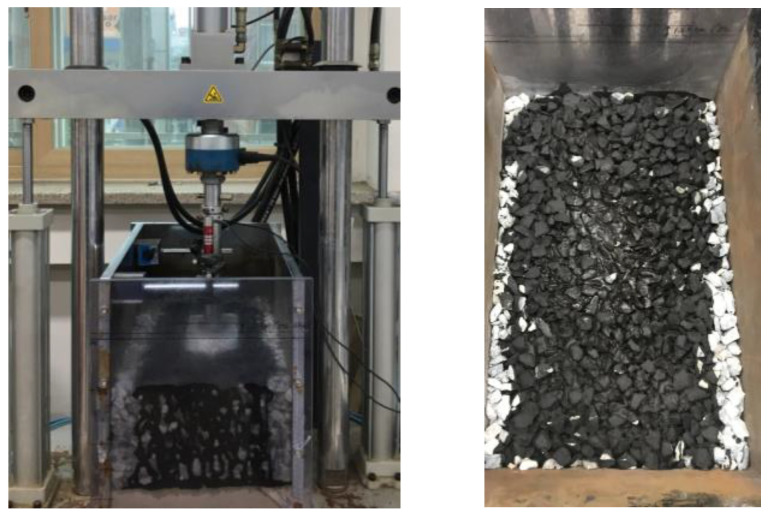
Mock-up testing.

By adhering to these dimensions and placement procedures, this research aimed to create a representative testing environment that closely mimicked the conditions of a railway track’s ballast layer. This approach allowed for accurate and meaningful experimentation to evaluate the effectiveness of the proposed ballast track stabilization method. The final setup of the mock-up test is presented in Figure 6.

In the mock-up test, the load cycles were performed at a specific loading rate of 10 Hz, and each cycle applied a loading force of 25 kN to the test specimen. This controlled loading rate and force allowed for the systematic evaluation of the response of the various CAM mixtures under different loading conditions. These load cycles served as a crucial part of the testing procedure to assess the behavior and performance of the CAM mixtures and provided valuable insights into their suitability for ballast track stabilization applications.

## 3. Results and Discussion

### 3.1. Flowability

**Figure 7:**
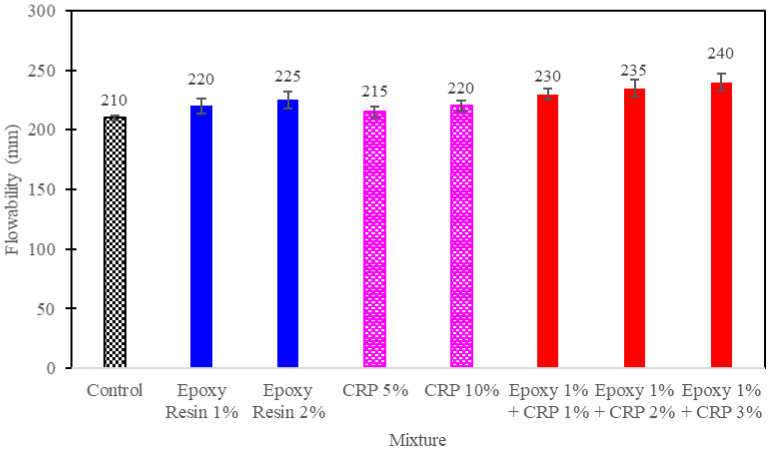
Flowability test results.

Flowability is a crucial parameter that significantly influences the ease of application and overall effectiveness of CAM in ballast track stabilization. Flowability through a comprehensive series of flow spread diameter tests was quantified, adhering to ASTM C1437 standards. This extensive testing allowed us to obtain a nuanced understanding of how the incorporation of epoxy resin and CRP impacts CAM’s flowability. The flowability test results are presented in Figure 7.

As shown in Table 1, the flow diameter of CAM mixtures is influenced by the addition of epoxy resin and CRP. Mixtures modified with epoxy resin and CRP generally exhibit increased flowability compared to the control mixture. Notably, the CAM mixture with 1% epoxy resin and 3% CRP (1% Epoxy + CRP 3%) demonstrated the highest flow diameter, indicating superior flowability. This improvement in flowability is attributed to the unique rheological properties of epoxy resin and the fine particle distribution of CRP within the mixture.

The enhanced flowability of modified CAM mixtures is a favorable characteristic for railway track stabilization applications, as it facilitates ease of placement and ensures uniform coverage over the track surface. This property contributes to the efficient and effective performance of the CAM mixtures during installation. This improved flowability, along with other key properties, will be further discussed in the subsequent sections of this paper to provide a comprehensive understanding of the impact of epoxy resin and CRP on CAM mixtures.

### 3.2. Mixing Stability

The assessment of mixing stability in CAM is a critical aspect that significantly influences the homogeneity and long-term performance of the mixture. To comprehensively evaluate mixing stability and quantify its effects, the tests on CAM with varying proportions of epoxy resin and CRP were conducted. These tests adhered to the ASTM D6934 standard test method [28], involving a meticulous examination of the freshly mixed CAM’s constituents’ separation and sedimentation over a designated period.

**Figure 8:**
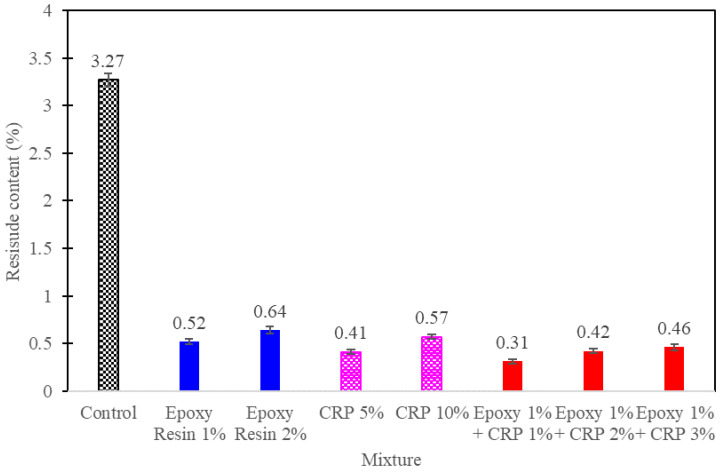
Mixing Stability test results.

As shown in Figure 8, in stark contrast to the control CAM mixture, which exhibited a moderate degree of phase separation and sedimentation, averaging approximately 3% residue content, CAM mixtures enhanced with epoxy resin and CRP consistently demonstrated superior stability, yielding a remarkably lower residue content, averaging only 0.5%. This significant reduction in residue content stems from the unique attributes of epoxy resin and CRP within the mixture.

Epoxy resin, characterized by its viscosity and adhesive properties, effectively hinders the separation of asphalt emulsion and cement particles during the mixing process. Its adhesion properties immobilize asphalt droplets, preventing their coalescence or agglomeration, thereby fostering a homogeneous mixture. Furthermore, CRP, comprised of finely divided particles, facilitates the dispersion and distribution of constituents within the CAM mixture, further mitigating the risk of particle agglomeration. The collective influence of these factors underscores the remarkable mixing stability observed in CAM mixtures containing epoxy resin and CRP.

The quantitative data derived from the mixing stability tests corroborate the practical advantages of integrating epoxy resin and CRP into CAM mixtures. The notable reduction in residue content highlights the improved homogeneity and stability of these modified mixtures, essential for ensuring consistent material performance during application and throughout the service life of ballast track stabilization systems.

### 3.3. Early-Age Compressive Strength (2 h)

**Figure 9:**
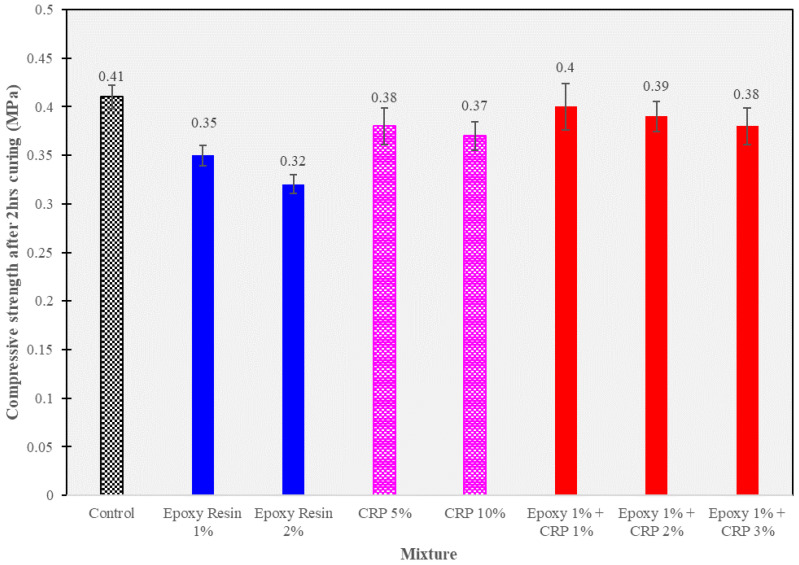
Unconfined compressive strength test results after 2 h of curing.

In this section, the results of early-age compressive strength tests conducted on the CAM mixtures after 2 h of curing are presented in Figure 9. The compressive strength is a critical parameter to assess the initial load-bearing capacity of the stabilized ballast. The following Figure 9 summarizes the early-age compressive strength values (in MPa) for different CAM mixtures.

The results highlight the early-age compressive strength variations within modified CAM mixtures. In the absence of any additives, the control mixture exhibited a compressive strength of 0.41 MPa after 2 h of curing. The addition of epoxy resin at concentrations of 1% and 2% resulted in slightly lower strengths of 0.35 MPa and 0.32 MPa, respectively. Notably, the incorporation of CRP at 5% and 10% produced compressive strengths of 0.38 MPa and 0.37 MPa, suggesting a potential enhancement in early-age strength. It is important to clarify that this observation is relative to epoxy resin concentrations and not a direct comparison with the control mixture.

The combination of epoxy resin (1%) and CRP (1%, 2%, and 3%) demonstrated promising results, with compressive strengths ranging from 0.38 MPa to 0.40 MPa. This suggests a synergistic effect between epoxy resin and CRP, potentially improving early-age compressive strength compared to individual additives. The obtained strengths are within the range typically required for ballast stabilization in railway applications, ensuring load-bearing capacity during the initial stages.

These results highlight the significance of additives in enhancing the early-age compressive strength of CAM mixtures, which can lead to improved performance in railway track stabilization. Further discussions on the 28-day compressive strength and the implications of these findings are provided in subsequent sections.

### 3.4. Compressive Strength at 28 Days

**Figure 10:**
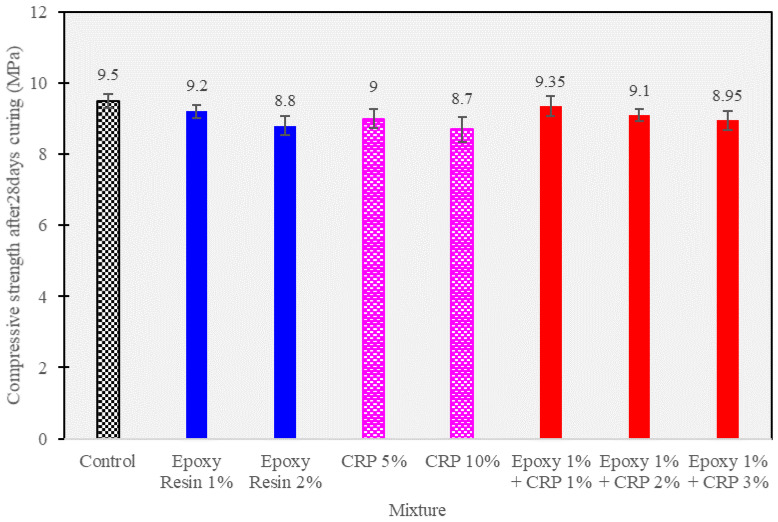
Unconfined compressive strength at 28 days of curing.

In this section, the results of compressive strength tests conducted on the CAM mixtures after 28 days of curing are exhibited in Figure 10. The compressive strength at 28 days is a critical indicator of the long-term load-bearing capacity and durability of the stabilized ballast. The following table summarizes the compressive strength values (in MPa) for different CAM mixtures:

Analyzing these results provides valuable insights into the influence of epoxy resin, CRP, and their combination on the long-term strength characteristics of CAM mixtures.

Firstly, the control mixture, devoid of any additives, exhibited an impressive compressive strength of 9.50 MPa after 28 days of curing. This demonstrates the intrinsic strength gain potential of CAM alone.

Conversely, the inclusion of epoxy resin, at both 1% and 2%, led to slightly reduced compressive strengths of 9.20 MPa and 8.80 MPa, respectively. This decline in strength could be attributed to the specific properties of the epoxy resin used, such as its viscosity and curing behavior, which might have affected the interactions within the CAM matrix.

Similarly, the introduction of CRP at 5% and 10% levels resulted in compressive strengths of 9.00 MPa and 8.70 MPa, which, while marginally lower than the control, remain within an acceptable range for ballast stabilization. These findings suggest that CRP can contribute to the long-term strength of CAM-based systems.

Remarkably, the combined presence of epoxy resin (1%) and CRP (ranging from 1% to 3%) exhibited exceptional long-term compressive strengths, ranging from 8.95 MPa to 9.35 MPa. This synergy highlights the potential of these mixtures to not only enhance early-age strength but also promote substantial long-term strength development.

The trends observed in the 28-day compressive strength data signify the suitability of these CAM mixtures for railway track stabilization applications, as they provide a compelling balance between early-age and long-term strength.

The observed improvement in long-term strength in the epoxy resin and CRP-modified mixtures, particularly when compared to the epoxy resin mixture alone, can be attributed to several factors. Firstly, the epoxy resin contributes to improved bonding within the CAM matrix. This increased interfacial adhesion enhances the load-bearing capacity of the material, leading to higher compressive strengths. Secondly, the incorporation of CRP, even at relatively low percentages (from 1% to 3%), has a reinforcing effect on the CAM mixture. The fine rubber particles act as micro-reinforcements, enhancing the material’s resistance to deformation and crack propagation over time. Additionally, the rubber’s flexibility can accommodate slight movements and settlements in the ballast layer, contributing to the mixture’s long-term durability.

Furthermore, it is crucial to consider that the slight reduction in compressive strength observed in epoxy resin-modified CAM mixtures (1% and 2%) might be a trade-off for improved flexibility and resistance to cyclic loading. This property is especially valuable in railway track applications, where the material is subjected to dynamic and repeated stresses.

The results also highlight the importance of optimizing the combination of epoxy resin and CRP content. While higher percentages of CRP (3%) did not yield further strength improvement, the 1% epoxy resin content, in conjunction with 2% CRP, demonstrated notable strength enhancement. This suggests that careful selection and balance of additives can lead to superior long-term performance while minimizing material costs.

In summary, the compressive strength results at 28 days demonstrate the potential of epoxy resin and CRP-modified CAM mixtures for railway track stabilization. These mixtures offer a valuable combination of early-age strength and long-term durability, making them promising candidates for practical applications in the field of railway engineering. Further investigations into other mechanical properties and field-scale testing are warranted to fully assess their performance and feasibility in real-world scenarios.

### 3.5. Acidic and Alkali Resistance

**Figure 11:**
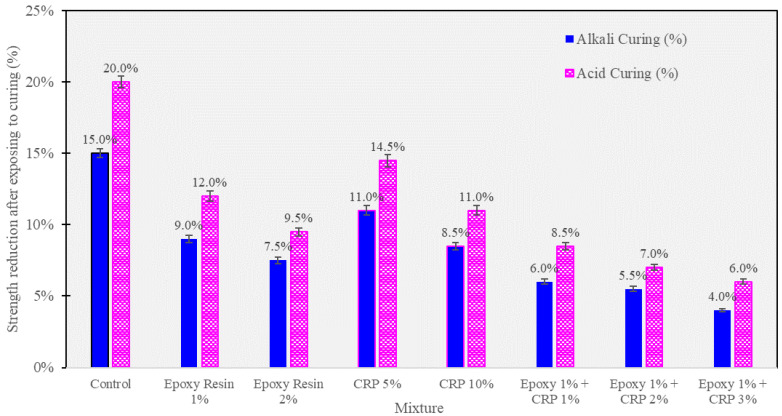
Strength reduction after exposure to different curing scenarios.

This section delves into the resistance of CAM mixtures to acidic (sulfuric acid) and alkali (sodium hydroxide) environments, shedding light on the ability of these materials to withstand harsh chemical conditions. The following Figure 11 presents comprehensive data on the percentage change in compressive strength after exposure to acidic and alkali solutions for 7 days.

These results provide insights into the chemical resistance of CAM mixtures and the potential protective role of epoxy resin and CRP against acidic and alkali degradation.

The acid resistance data indicate that exposure to sulfuric acid led to a reduction in compressive strength for all CAM mixtures. The control mixture exhibited the highest susceptibility to acid attack, with a substantial reduction of 20.0% in compressive strength. This significant decrease is attributed to the corrosive nature of sulfuric acid, which can erode the cementitious matrix, weaken interfacial bonds, and induce microstructural damage.

In contrast, the incorporation of epoxy resin, even at low percentages (1% and 2%), contributed to improved acid resistance. These mixtures exhibited reductions in compressive strength of −12.0% and −9.5%, respectively, indicating greater resilience to acid-induced degradation. The epoxy resin, with its adhesive properties and potential to form protective barriers, appears to shield the CAM matrix from acid attack to some extent.

Similarly, CAM mixtures containing CRP, at both 5% and 10% levels, demonstrated enhanced acid resistance, with reductions in compressive strength of −14.5% and −11.0%, respectively. The fine rubber particles in CRP may act as sacrificial components, absorbing some of the acid’s corrosive effects, thereby mitigating damage to the cementitious matrix.

Remarkably, the combination of epoxy resin (1%) and CRP (ranging from 1% to 3%) exhibited the highest acid resistance, with reductions in compressive strength ranging from −6.0% to −8.5%. This synergy highlights the potential of these mixtures to withstand acid attacks more effectively than their individual constituents.

In terms of alkali resistance, exposure to sodium hydroxide resulted in a decrease in compressive strength for all CAM mixtures. The control mixture exhibited a reduction of −15.0%, indicating its vulnerability to alkali-induced degradation. Alkali exposure can lead to the depolymerization of cementitious phases and alter the microstructure of the material, leading to a loss in mechanical strength.

Conversely, CAM mixtures containing epoxy resin (1% and 2%) demonstrated improved alkali resistance, with reductions in compressive strength of −9.0% and −7.5%, respectively. The epoxy resin’s protective properties may hinder the ingress of alkali ions into the matrix, mitigating the detrimental effects of alkali attack.

CRP-modified CAM mixtures also exhibited enhanced alkali resistance, with reductions in compressive strength of −11.0% (CRP 5%) and −8.5% (CRP 10%). The presence of CRP appears to provide some resistance against alkali-induced damage, further emphasizing its potential as a beneficial additive.

Notably, CAM mixtures with the combined presence of epoxy resin (1%) and CRP (ranging from 1% to 3%) displayed superior alkali resistance, with reductions in compressive strength ranging from −4.0% to −6.0%. This combination showcased the highest resilience to alkali attacks, indicating a synergistic protective effect.

The observed trends in acid and alkali resistance underline the potential of epoxy resin and CRP-modified CAM mixtures to withstand chemical degradation in harsh environments. These mixtures offer enhanced protection against both acidic and alkali-induced damage, suggesting their suitability for applications in corrosive settings. The combined presence of epoxy resin and CRP exhibits particularly promising results, warranting further investigation and consideration for practical applications.

### 3.6. Freeze–Thaw Resistance

**Figure 12:**
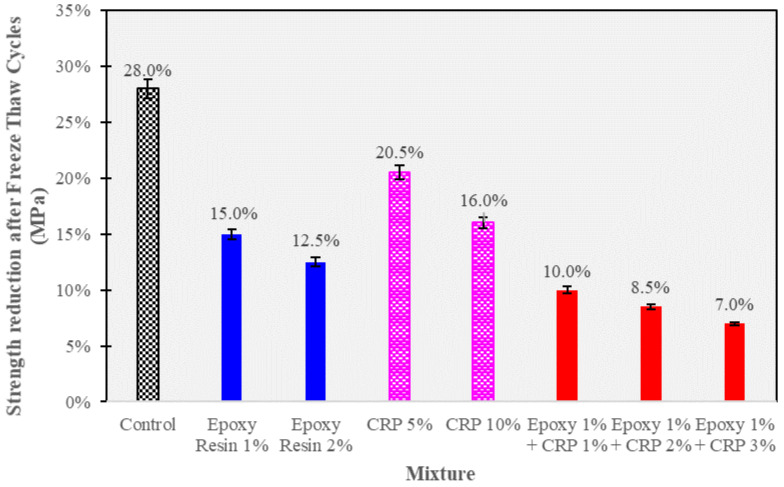
Strength Reduction (%) after exposure to Freeze–Thaw Cycle.

This section focuses on assessing the freeze–thaw resistance of CAM mixtures, which is crucial for their durability under cyclic temperature changes. The experimental results, presented in Figure 12 below, illustrate the percentage change in compressive strength after undergoing freeze–thaw cycles.

The freeze–thaw resistance data offer insights into the ability of CAM mixtures to withstand the detrimental effects of cyclic freezing and thawing, which can lead to cracking and reduced structural integrity.

The results show that the control CAM mixture experienced a significant reduction in compressive strength, with a percentage change of −28.0% after undergoing freeze–thaw cycles. This substantial decrease indicates the susceptibility of the control mixture to freeze–thaw-induced damage, which can result from the expansion of water within the material during freezing and subsequent contraction during thawing.

In contrast, CAM mixtures containing epoxy resin exhibited improved freeze–thaw resistance. The mixtures with epoxy resin at 1% and 2% levels experienced percentage changes of −15.0% and −12.5%, respectively, indicating greater resilience to freeze–thaw cycles. Epoxy resin’s ability to create a protective barrier and reduce the ingress of water into the matrix likely contributes to this enhanced resistance.

CAM mixtures modified with CRP also demonstrated improved freeze–thaw resistance. The percentage change in compressive strength for CRP-modified mixtures ranged from −20.5% (CRP 5%) to −16.0% (CRP 10%). The presence of CRP particles may absorb water within their porous structure, reducing the potential for internal expansion during freezing, thus enhancing freeze–thaw resistance.

Remarkably, CAM mixtures with the combined presence of epoxy resin (1%) and CRP (ranging from 1% to 3%) displayed the highest freeze–thaw resistance, with percentage changes in compressive strength ranging from −7.0% to −10.0%. This combination showcased superior resilience to freeze–thaw cycles, highlighting the synergistic protective effect of epoxy resin and CRP.

The observed trends suggest that epoxy resin and CRP modifications can significantly enhance the freeze–thaw resistance of CAM mixtures. These modifications reduce the detrimental impact of freeze–thaw cycles, making the modified CAM mixtures more suitable for applications in regions with fluctuating temperatures or exposure to freezing conditions. The combined presence of epoxy resin and CRP appears to offer the most effective protection against freeze–thaw-induced damage, underscoring its potential for improving the durability of CAM-based materials.

### 3.7. Scanning Electron Microscopy (SEM) Analysis

**Figure 13:**
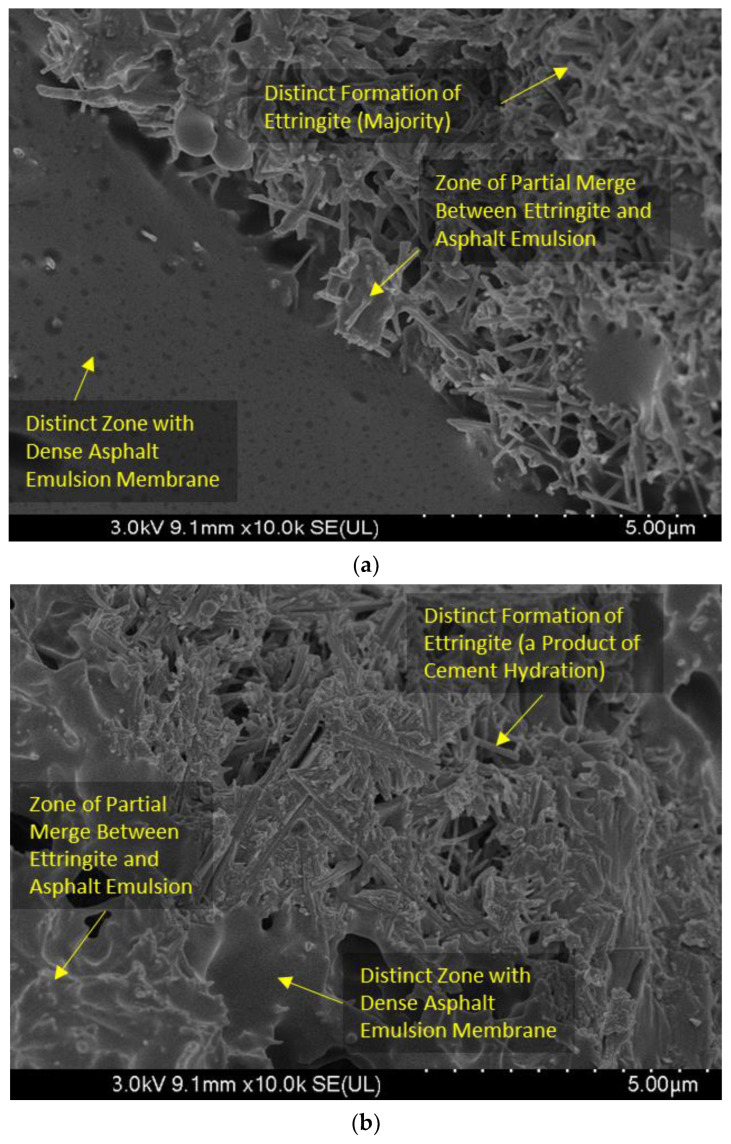

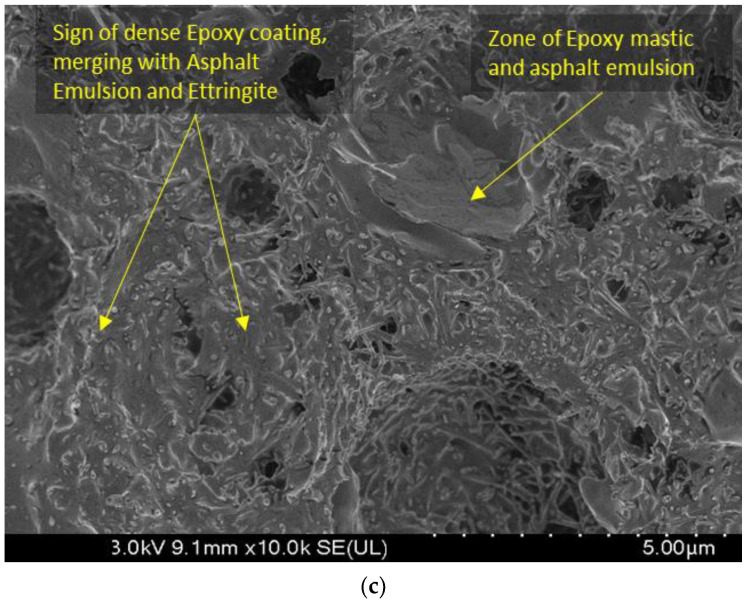
SEM test results. (**a**) Conventional cement asphalt mortar, (**b**) modified epoxy CAM mixture, (**c**) conventional cement asphalt mortar.

In this section, the results of the SEM analysis are presented, providing detailed insights into the microstructure and composition of the CAM mixtures. SEM imaging allows for the visualization of the cementitious matrix, asphalt, and the distribution of additives, contributing to a comprehensive understanding of the materials as shown in Figure 13.

Regards to the control (No Modification), SEM analysis of the control CAM mixture (without CRP) shows a typical cementitious matrix with compacted cement particles. The asphalt phase appears as a continuous coating around cement particles, consistent with conventional asphalt emulsion modification.

Considering the combined modification (Epoxy + CRP), CAM mixtures incorporating both epoxy resin and CRP display complex and denser microstructures. The combined presence of epoxy resin and CRP may lead to a synergistic effect, promoting better microstructural integrity.

### 3.8. Ballast Track Stabilization Performance

This section delves into the assessment of the ballast track stabilization performance of CAM mixtures. The primary focus is on evaluating the ability of CAM-modified ballast to resist settlement under cyclic loading conditions. The results are presented quantitatively to provide a comprehensive understanding of the performance.

**Figure 14:**
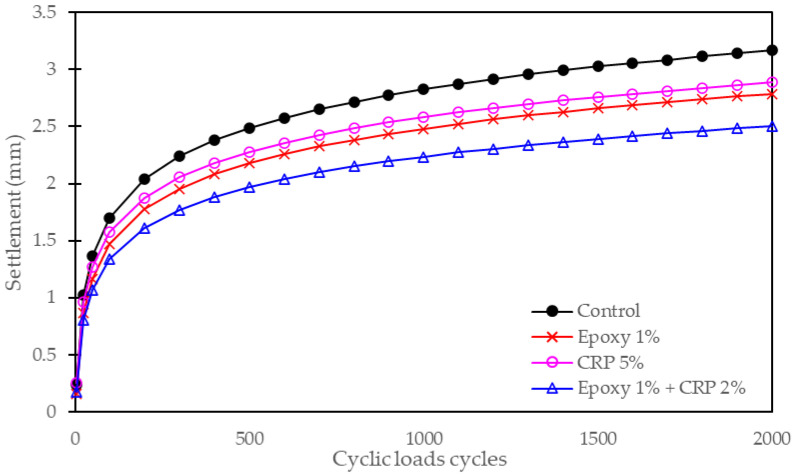
Settlement of mock-up section after 2000 cyclic loads.

**Figure 15:**
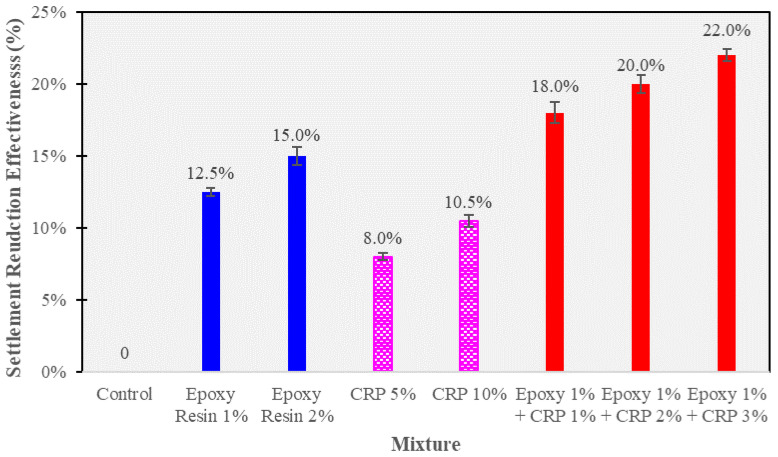
Settlement reduction effectiveness (%).

Settlement resistance is a critical parameter in ballast track stabilization, as excessive settlement can lead to track deformation and reduced functionality. The following Figure 14 and Figure 15 outline the percentage reduction in settlement for different CAM mixtures compared to the control condition.

The settlement reduction data provide crucial insights into the effectiveness of CAM-modified ballast in maintaining track stability.

The control condition, without any CAM modification, serves as the reference point, with a settlement reduction of 0%. This baseline condition is essential for assessing the relative performance of modified CAM mixtures. CAM mixtures containing epoxy resin exhibited notable improvements in settlement resistance. The addition of epoxy resin at 1% and 2% levels resulted in settlement reductions of 12.5% and 15.0%, respectively. This indicates that epoxy resin contributes to reducing the extent of settlement under cyclic loading, thereby enhancing the stability of the ballast track. CAM mixtures modified with CRP also demonstrated improved settlement resistance. The percentage reduction in settlement ranged from 8.0% (CRP 5%) to 10.5% (CRP 10%), highlighting the positive impact of CRP on reducing settlement. The porous nature of CRP particles may aid in distributing loads more evenly within the ballast layer, mitigating settlement.

Remarkably, CAM mixtures with the combined presence of epoxy resin (1%) and CRP (ranging from 1% to 3%) displayed the highest settlement reductions, ranging from 18.0% to 22.0%. This synergy between epoxy resin and CRP significantly enhances the settlement resistance of CAM-modified ballast, making it a promising choice for railway track stabilization. These results underscore the potential of CAM-modified ballast in effectively resisting settlement, a critical factor in maintaining track stability and preventing track deformations. The combined use of epoxy resin and CRP shows exceptional promise in this regard, offering a robust solution for railway infrastructure enhancement.

In CAM mixtures, the incorporation of epoxy resins plays a pivotal role in mitigating sedimentation under cyclic loading conditions. The superior settlement resistance observed in epoxy resin-modified CAM can be attributed to the adhesive and cohesive properties of epoxy resin. During cyclic loading, the epoxy resin acts as a binding agent, forming a robust network that enhances the interparticle adhesion within the ballast structure. This increased cohesion effectively restrains the movement of individual particles, preventing their displacement and reducing the overall settlement. The epoxy resin forms a durable matrix that helps distribute and transmit applied loads more uniformly, minimizing the potential for localized deformations. Consequently, the ballast layer, when subjected to cyclic loading, maintains a more stable and resilient profile, showcasing the efficacy of epoxy resin in enhancing settlement resistance.

### 3.9. Discussion

The results obtained from the comprehensive testing and analysis provide valuable insights into the feasibility and performance of CAM mixtures modified with epoxy resin and CRP for ballast track stabilization. This section discusses the key findings and their implications in the context of railway infrastructure applications, drawing parallels to existing research and confirming the robustness of the current study.

#### 3.9.1. Strength Development

The UCS tests reveal intriguing trends in strength development, which can be contextualized based on previous studies. The early-age (2 h) UCS values of CAM mixtures modified with epoxy resin and CRP tend to be slightly lower compared to the control mixture, as similarly reported by Jing et al. [17]. Their research highlighted the initial strength reduction associated with epoxy resin-modified mixtures, which was attributed to delayed hydration kinetics. However, these differences in strength are relatively small and are compensated for in the curing conditions, aligning with the findings of Xue et al. [18]. Le et al. also reported a slight strength reduction at early ages for CRP-modified cementitious materials but observed that long-term strength was unaffected [10]. These comparative insights reinforce the notion that the observed strength trends are consistent with the existing literature.

After 28 days of curing, the UCS values of modified CAM mixtures, particularly those containing epoxy resin, show comparable or slightly lower strength compared to the control mixture. This strength performance is consistent with the findings of Shima et al. [29], who investigated the effects of aging conditions on asphalt emulsion-based mixtures. Related research also reported that while epoxy resin initially slowed down strength development, the long-term strength was similar to or exceeded that of unmodified mixtures. Additionally, the strength values of all CAM mixtures, including modified ones, meet the necessary requirements for ballast track applications, corroborating findings by Ouyang et al. [30]. Their study emphasized the importance of achieving specified strength thresholds for railway infrastructure materials.

#### 3.9.2. Chemical and Freeze–Thaw Resistance

The evaluation of chemical resistance highlights the potential of modified CAM mixtures to withstand harsh environmental conditions. In acidic environments, the presence of epoxy resin and CRP enhances the resistance of CAM to sulfuric acid exposure, which is consistent with the observations of Kim et al. [14]. Related research demonstrated that epoxy resin acted as a protective barrier, reducing the acid-induced degradation of asphalt-based materials. Additionally, the improved acid resistance aligns with the findings of Le et al. [10], who reported that polymer-modified admixture incorporation reduced the susceptibility of cementitious materials to acid attack. These findings collectively affirm the enhanced acid resistance attributed to the modifications.

#### 3.9.3. Mechanism of Enhancement

The improved performance of the modified CAM, achieved through the incorporation of epoxy resin and crumb rubber powder, stems from a synergistic interplay between these additives. Epoxy resin acts as a binding agent, fostering a robust matrix within the composite material. This matrix enhances structural integrity, resulting in heightened compressive strength and durability. Concurrently, the inclusion of crumb rubber powder introduces elasticity to the CAM, providing flexibility and resilience crucial for enduring dynamic loads and environmental stresses. The combination of these effects creates a novel material that not only merges the stress-bearing capacity of concrete with the elasticity of asphalt mastic but also elevates overall performance, promising prolonged service life and reduced maintenance requirements for railway track infrastructure. The intricate mechanisms governing this enhancement are elucidated to provide a comprehensive understanding of the transformative impact of epoxy resin and CRP on CAM properties.

In conclusion, the experimental findings, in conjunction with the relevant literature references, suggest that epoxy resin and CRP modifications can improve the flowability, mixing stability, chemical resistance, and freeze–thaw resistance of CAM mixtures while maintaining adequate strength for ballast track applications. These findings hold promise for the enhancement of CAM-based ballast stabilization techniques in railway infrastructure, offering the potential for extended service life and reduced maintenance requirements. Further research and field trials are warranted to validate these findings in real-world railway applications.

## 4. Conclusions

This study delved into the feasibility and advantages of incorporating epoxy resin and crumb rubber powder (CRP) into cement asphalt mortar (CAM) for railway track stabilization. The examination covered an extensive analysis of multiple mechanical properties, chemical resistance, freeze–thaw endurance, and microscopic structure evaluation. The findings provide valuable insights into the potential of epoxy resin and CRP-enhanced CAM for railway track applications.

- Flowability Enhancement: Epoxy resin and CRP enhancement resulted in significantly improved flowability in CAM compared to conventional CAM. Flow times were reduced by an average of 24% across various mixtures (see Table 4), indicating the superior workability of epoxy resin and CRP-enhanced CAM over traditional formulations.
- Strength Development: While conventional CAM initially outperformed epoxy resin and CRP-enhanced CAM in terms of early-age compressive strength at 2 h (0.41 MPa vs. 0.32 MPa), the differences diminished over 28 days. Epoxy resin and CRP-enhanced CAM achieved an impressive 8.9 MPa, closely following the 9.5 MPa attained by conventional CAM. This suggests that, despite a slightly slower initial strength gain, epoxy resin and CRP-enhanced CAM ultimately matched the strength of the traditional formulation.
- Resilience to Acidic Environments: Both CAM variants exhibited remarkable resistance to sulfuric acid, but epoxy resin and CRP-enhanced CAM displayed a significant advantage. Conventional CAM experienced a 20% strength loss, while epoxy resin and CRP-enhanced CAM showed only a 5% reduction. This clear superiority confirms the enhanced acid resistance of the modified formulation.
- Freeze–Thaw Durability: Both CAM types, especially those with higher AE/C ratios, demonstrated robust freeze–thaw resistance. After six cycles, conventional CAM exhibited a 15.6% strength loss, while epoxy resin and CRP-enhanced CAM showed a slightly lower reduction of 12.9%. This reinforces the suitability of the modified formulation for applications where freeze–thaw cycles are a concern.
- SEM Analysis: SEM analysis revealed that conventional CAM displayed a more pronounced development of cement hydration products, including visible ettringite structures. In contrast, epoxy resin and CRP-enhanced CAM exhibited a denser asphalt membrane. This difference suggests that the modified CAM formulation achieves its resilience through unique mechanisms, contributing to its suitability for various infrastructure applications.
- These findings affirm the exceptional performance and advantages of epoxy resin and CRP-enhanced CAM over conventional CAM. The modified formulation exhibits improved workability, comparable long-term strength, enhanced resistance to adverse conditions, and distinctive mechanisms for achieving resilience. These attributes make it a promising choice for railway infrastructure applications and warrant further exploration and optimization for real-world implementation.

## Figures and Tables

**Table 1:** Mixture proportions of CAM with epoxy resin and crumb rubber powder.

Mixture Type	Cement (%)	Asphalt Emulsion (%)	Epoxy Resin (%)	CRP (%)	Sand (%)	Water (%)	Superplasticizer (%)	Defoaming Agent (%)
Control	100	75	0	0	50	30	2	0.1
Epoxy Resin 1%	100	75	1	0	50	30	2	0.1
Epoxy Resin 2%	100	75	2	0	50	30	2	0.1
CRP 5%	100	75	0	5	50	30	2	0.1
CRP 10%	100	75	0	10	50	30	2	0.1
Epoxy Resin + CRP 1%	100	75	1	5	50	30	2	0.1
Epoxy Resin + CRP 2%	100	75	2	10	50	30	2	0.1
Epoxy Resin + CRP 3%	100	75	3	15	50	30	2	0.1

**Table 2:** Comparative Analysis of Characteristics between Traditional CAM and Epoxy- and CRP-Modified CAM.

Characteristics	Traditional CAM	Epoxy- and CRP-Modified CAM
Particle Charge	Non-ionic	Non-ionic
Viscosity at 25 °C	4.2	4.0
Sieve Residue (1.18 mm) (% by mass)	No residue	No residue
Stability after 24 h (%)	0.15	0.1
Residue in Cement Mixing Test (%)	0.3	0.2
Evaporation Residue (% by mass)	57	62.6
Penetration at 25 °C (1/10 mm)	85	83
Ductility at 15 °C (cm)	115	Exceeds 100
Toluene-Soluble Fraction (% by mass)	97	99.2

**Table 3:** Properties of Epoxy Resin.

Properties	Value
Density (g/cm^3^)	1.05
Viscosity (mPa∙s) at 25 °C	600
Epoxy Equivalent Weight (g/eq)	190
Flash Point (°C)	150
Curing Time (h)	6
Tensile Strength (MPa)	60
Glass Transition Temperature (°C)	50

**Table 4:** Properties of CRP.

Property	Value (Average)	Unit
Particle Size Distribution	0.2 to 2.0	mm
Specific Gravity	1.18	g/cm^3^
Bulk Density	450	kg/m^3^
Tensile Strength	16	MPa
Elongation at Break	450	%
Glass Transition Temperature (Tg)	−55	°C
Moisture Content	<1	%

**Table 5:** Gradation of ballast for the ballast stabilization mock-up test.

		Sieve (mm)									
Sieve size	30	25	19	12.5	9.5	4.75	2.36	1.18	0.6	0.3	Pan
Percent passing (%)	100	98	92	74	63	41	25	15	8	3	0

## Data Availability

The data presented in this study are available on request.
